# Dual blockade of BRD4 and ATR/WEE1 pathways exploits *ARID1A* loss in clear cell ovarian cancer

**DOI:** 10.21203/rs.3.rs-3314138/v1

**Published:** 2023-09-27

**Authors:** Yasuto Kinose, Haineng Xu, Hyoung Kim, Sushil Kumar, Xiaoyin Shan, Erin George, Xiaolei Wang, Sergey Medvedev, Benjamin Ferman, Sarah B. Gitto, Margaret Whicker, Kurt D’Andrea, Bradley Wubbenhorst, Dorothy Hallberg, Mark O’Connor, Lauren E. Schwartz, Wei-Ting Hwang, Katherine L Nathanson, Gordon B. Mills, Victor E. Velculescu, Tian-Li Wang, Eric J. Brown, Ronny Drapkin, Fiona Simpkins

**Affiliations:** 1 Penn Ovarian Cancer Research Center, Perelman School of Medicine, University of Pennsylvania, Philadelphia, PA; 2 Department of Obstetrics and Gynecology, Division of Gynecologic Oncology, Hospital of the University of Pennsylvania, Philadelphia, PA; 3 Department of Pathology and Laboratory Medicine, Abramson Cancer Center, Perelman School of Medicine, University of Pennsylvania, Philadelphia, PA, 19104, USA; 4 Department of Medicine, Division of Translational Medicine and Human Genetics, Abramson Cancer Center, Perelman School of Medicine, University of Pennsylvania, Philadelphia, PA 19104, USA.; 5 The Sidney Kimmel Comprehensive Cancer Center, Johns Hopkins University School of Medicine, Baltimore, MD; 6 AstraZeneca, R&D Oncology, Cambridge, United Kingdom; 7 Department of Pathology, Hospital of the University of Pennsylvania, Philadelphia, PA 19104; 8 Department of Biostatistics, Epidemiology and Informatics, University of Pennsylvania, Philadelphia, PA 19104, USA; 9 Division of Oncological Sciences Knight Cancer Institute, Oregon Health and Science University, Portland, OR; 10 Department of Cancer Biology and the Abramson Family Cancer Research Institute, Perelman School of Medicine, University of Pennsylvania, Philadelphia, PA 19104, USA

**Keywords:** clear cell ovarian cancer, ARID1A, BRD4, ATR, WEE1, DNA damage response

## Abstract

*ARID1A,* an epigenetic tumor suppressor, is the most common gene mutation in clear-cell ovarian cancers (CCOCs). CCOCs are often resistant to standard chemotherapy and lack effective therapies. We hypothesized that *ARID1A* loss would increase CCOC cell dependency on chromatin remodeling and DNA repair pathways for survival. We demonstrate that combining BRD4 inhibitor (BRD4i) with DNA damage response inhibitors (ATR or WEE1 inhibitors; e.g. BRD4i-ATRi) was synergistic at low doses leading to decreased survival, and colony formation in CCOC in an *ARID1A* dependent manner. BRD4i-ATRi caused significant tumor regression and increased overall survival in *ARID1A*^MUT^ but not *ARID1A*^WT^ patient-derived xenografts. Combination BRD4i-ATRi significantly increased γH2AX, and decreased RAD51 foci and BRCA1 expression, suggesting decreased ability to repair DNA double-strand-breaks (DSBs) by homologous-recombination in *ARID1A*^MUT^ cells, and these effects were greater than monotherapies. These studies demonstrate BRD4i-ATRi is an effective treatment strategy that capitalizes on synthetic lethality with *ARID1A* loss in CCOC.

## Introduction

Ovarian cancer is a heterogeneous disease with multiple histological subtypes^1^. There is a global consensus on the necessity of defining treatment strategies for ovarian cancer by histologic subtype. Even though less common in the US, clear cell ovarian cancer (CCOC) represents more than 25% of all ovarian cancers in Asia including Japan, Taiwan, and Singapore^2^. CCOC is one of the most challenging subtypes to treat as it is relatively insensitive to standard of care chemotherapies and is thus associated with a worse prognosis than more common subtypes, such as high-grade serous ovarian cancer (HGSOC)^3–5^. Response rates to standard front-line chemotherapy for patients with measurable residual tumor after debulking surgery with advanced disease are only 11.1% for CCOC compared to 72.5% for HGSOC^5^. Even more challenging is the lack of effective therapies for platinum-resistant recurrent CCOC with response rates of about 1% with various chemotherapy regimens^6^. Strategies to identify more effective therapeutic options for CCOC are clearly a clinical unmet need.

CCOCs demonstrate unique genetic alterations (e.g., *ARID1A*, *PIK3CA*, *PPP2R1A*, *KRAS*, *PTEN*) which may be exploited by targeted therapies^7,8^. Despite distinct genomic/molecular alterations in CCOC, they are currently treated similarly to HGSOC with standard platinum-based chemotherapy. Not surprisingly, treatment results are poor as described above^3–6,9^. Unlike HGSOC which is thought to originate from the distal fallopian tube^10^, CCOC is thought to arise from displaced endometriosis, further supporting a unique molecular landscape^11^. Additionally, CCOCs represent a heterogeneous disease at the genomic level despite having similar histological features^12^. Based on the transcriptome profiling of 212 primary tumors, Bolton et al. identified two distinct molecular subgroups for CCOCs, the first driven by *ARID1A* mutations (included those with *P1K3CA, TERT* mutations) and the second, with TP53 mutations (including mesenchymal differentiation and immune –related pathways). These subgroups each have distinct clinical outcomes, with the *TP53* subgroup fairing worse, and exhibiting potentially unique therapy responsiveness^13,14^. Recent gene interaction analysis and functional assessment in CCOCs revealed that mutated genes were clustered into groups related to chromatin remodeling, DNA repair, cell cycle checkpoint, and cytoskeletal organization^15^. Integrated analyses uncovered that frequently mutated or amplified/deleted genes were involved in the KRAS/PI3K (82%) and MYC/retinoblastoma (RB) (75%) pathways as well as the critical chromatin remodeling SWI/SNF complex (85%)^15^. *ARID1A* is the most prevalent mutation in CCOC, with more than 50% of all CCOC tumors harboring this mutation^16^. *ARID1A* is a member of the SWI/SNF complex, with family members having helicase and ATPase activities which also regulate transcription of a subset of genes by altering the chromatin structure around those genes. This complex also has a major role in the repair of DNA lesions by directly facilitating DNA accessibility on the chromatin or indirectly by facilitating the functions of DNA repair proteins (e.g. p53, BRCA1, ATR, and Fanconi anemia proteins)^17,18^. In addition, ARID1A maintains genome stability by protecting telomere cohesion and mutations or inactivation of ARID1A causes DNA damage^19^. The SWI/SNF complex is required for dozens of processes that are critical for cell cycle checkpoint control and differentiation^17^. Mutations, translocations and deletions involving various subunits of the SWI/SNF complex were found in ~20% of all human cancers, with *ARID1A* being the most frequently mutated member^20,21^. Recent studies show that SWI/SNF-mutant cancers depend on residual SWI/SNF complexes for their aberrant growth, thus revealing synthetic lethal interactions that could be exploited for therapeutic purposes^22,23^. Finally, while CCOCs exhibit unique genomic alterations as well as activation of various signaling pathways, including PI3K/AKT/mTOR, VEGF, HNF-1β, and IL-6/STAT3, no novel molecular-targeted therapies have yet been developed for this refractory subtype of ovarian cancer^7^.

Recently, several selective small molecule inhibitors influencing chromatin-modifying proteins have been developed as first-in-class cancer therapies^24,25^. Among them, Bromodomain and extraterminal domain (BET)-family protein inhibitors (BETi; JQ-1, iBET-762, AZD5153, and ZEN3694; clinicaltrials.gov) are being pursued in early clinical trials showing tolerability and anti-tumor activity for various types of cancers including HGSOC^25–29^. BETi bind to the bromodomain of BET proteins (predominantly BRD4) and prevent their interactions with acetylated histones thereby inhibiting the transcription of genes which are very important in tumorigenesis such as *MYC*, Receptor Tyrosine Kinases and downstream effectors such as *MTOR*^25,30^. CCOC and endometrioid ovarian cancer have increased expression of *c-MYC* by immunohistochemistry (IHC)^31^. BETi exhibit selectivity for tumor cells by preferentially binding to super-enhancers, noncoding regions of DNA critical for the transcription of genes that determine a cell's identity^21,24^. BRD4 also plays an important role in regulating the expression of genes required for M to early G1 phase transition. BRD4 recruits P-TEFb to chromosomes at late mitosis to promote G1 gene expression and cell cycle progression. Knocking down BRD4 leads to G1 cell cycle arrest and apoptosis^32^. Loss of *ARID1A* sensitizes breast cancer cells to BET inhibition^33^, and most *ARID1A* mutated (*ARID1A*^MUT^) ovarian clear cell carcinomas showed relative sensitivity to BETi compared with *ARID1A* wild type (*ARID1A*^WT^) *in vitro* and inhibited tumor growth *in vivo* as monotherapy^34^. Although BETi have strong and rational indication for *ARID1A*^MUT^ CCOC, concerns remain about the limited single-agent efficacy in the clinic^24^. Given cancers ultimately develop resistance to most monotherapy approaches, combination therapy is a strategy to potentially prevent emergence of resistance, and increase durability of responses^35^.

ARID1A-depleted cells have impaired G2/M checkpoint initiation and maintenance^36^. When DNA double strand breaks (DSB) are induced, ARID1A is recruited via its interaction with DNA damage checkpoint kinase ataxia telangiectasia and Rad3-related (ATR). It is required for DSB-induced ATR activation and promotes DSB end resection, leading to the sensitivity to PARP inhibitors (PARPi) in *ARID1A* deficient cancer cells *in vitro* and *in vivo*^36^. *ARID1A* deficiency also results in topoisomerase 2A and cell cycle defects, which cause an increased reliance on ATR checkpoint activity, and ATR inhibitors (ATRi) are synthetic lethal and active in *ARID1A* mutant tumors preclinically^37^. WEE1 tyrosine kinase is another key G2 cell cycle checkpoint regulator that arrests cells in G2, allowing for DNA repair by homologous recombination^38,39^. WEE1 inhibition abrogates the G2 checkpoint, and triggers premature cell cycle entry into mitosis with resulting mitotic catastrophe leading to cell death^38^. Given that *ARID1A*^MUT^ cancers are known to have defects in S and G2/M, WEE1 inhibitors (WEE1i) are also a rationale therapeutic approach to exploit *ARID1A*^MUT^ CCOC^36,37^. ATR and WEE1 inhibitors are currently being evaluated in phase I/II clinical trials as monotherapy and in combination with radiation or cytotoxic chemotherapy^40–44^. Considering effects of ATR/WEE1 inhibitors combined with vulnerabilities of *ARID1A*^MUT^ tumors induced by aberrations in DNA damage repair (DDR), ATRi and WEE1i are rational candidates for combination with BETi ^37,45^.

In this study, we found that BETi, ATRi and WEE1i were the most active monotherapies in *ARID1A*^MUT^ cells compared to *ARID1A*^WT^ cells in a drug screen. We found that low dose BRD4 inhibition (BRD4i) in combination with DNA damage response inhibitors (DDRi, BRD4i-ATRi or BRD4i-WEE1i) were synergistic in decreasing cell viability and colony formation in *ARID1A*^MUT^ cells compared to *ARID1A*^WT^ cells. Combination of BRD4i-ATRi led to significant tumor regression and increased overall survival comparing to standard chemotherapy or monotherapies in *ARID1A*^MUT^ CCOC patient-derived xenograft (PDX) models with less effect in *ARID1A*^WT^ PDXs. The BRD4i-ATRi combination induced G1 arrest, decreased homologous recombination (HR) regulators such as RAD51 and BRCA1 expression, leading to increased DNA DSB and cell apoptosis in *ARID1A*^MUT^ cells compared to wild type cells. Our studies identify a novel drug combination capitalizing on *ARID1A* mutations common in CCOC that is highly effective and tolerable, warranting evaluation in the clinic.

## Results

### Combined inhibition of BRD4 with ATR/WEE1 is synergistic in *ARID1A* mutant clear cell ovarian cancer cells.

To discover new effective treatments for CCOCs, we evaluated targeted therapies and current standard-of-care chemotherapies (carboplatin and paclitaxel) in a drug screen panel in 6 CCOC lines (*ARID1A*^*WT*^: ES-2, *ARID1A*^MUT^: TOV21G, OVTOKO, OVMANA, JHOC-9, and OVISE) (Supplementary Fig. 1A–B). Consistent with platinum-resistance, a clinical feature of CCOC, 5 out of 6 lines demonstrated an IC_50_ for carboplatin > 5 µg/ml (Supplementary Fig. 1A–B)^6^. At clinically comparable doses, BET inhibitors, BRD4i (AZD5153, 0.6 µM) and JQ1 (0.8 µM) followed by DNA damage response (DDR) inhibitors, WEE1i (AZD1775) and ATRi (AZD6738) where the most active in decreasing viability in *ARID1A* mutant compared to *ARID1A* wild type CCOCs. Chemotherapy (carboplatin and paclitaxel), PARPi (olaparib), DNA methyl transferase inhibitors (Decitabine), PI3K inhibitors (BKM120), EZH2 inhibitors (GSK126, EPZ6438) were less effective as measured by MTT assay (Supplementary Fig. 1A–B). Although DDR inhibitors (DDRi) and BET inhibitors are more effective in decreasing viability than chemotherapy and other agents (including inhibitors to PI3K, PARP, EZH2, DNMT), their monotherapy efficacy could potentially be further optimized.

**Figure 1.**
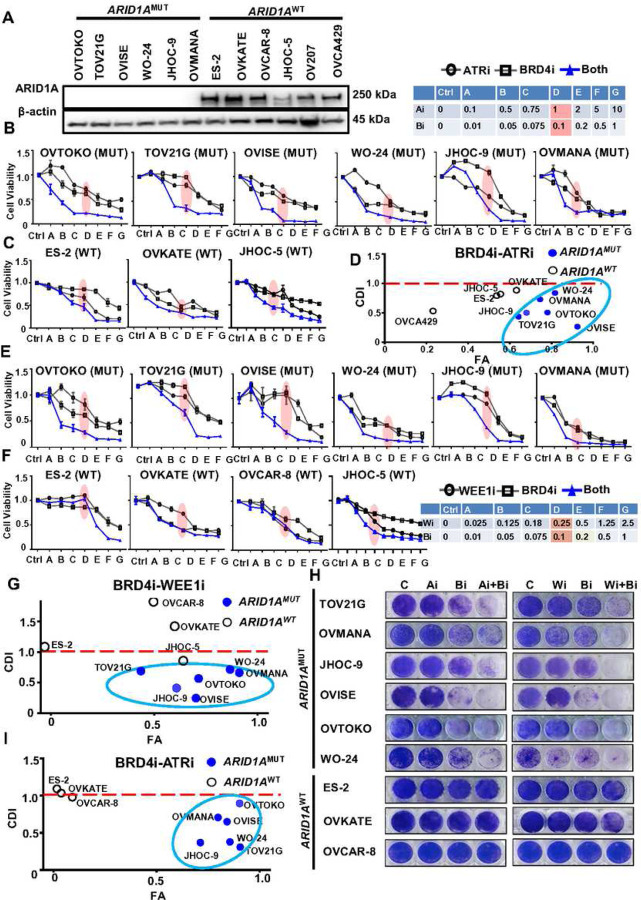
Combination inhibition of BRD4 and DNA damage response are synergistic in inhibiting ovarian cancer depending on *ARID1A* mutation status. (A) Western blot detection of ARID1A expression in gynecologic cancer lines. (B-C) Cell viability detection of BRD4i-ATRi combination at the indicated doses in *ARID1A*^MUT^ (B) and *ARID1A*^WT^ (C) cell lines. Cells were treated with monotherapy or drugs combination for 5 days (n = 3–6; mean ± SD). The dosage combination with best synergy (BRD4i: 0.1µM, ATRi 1µM) was highlighted in Red. (D) Comparison of BRD4i-ATRi synergy in *ARID1A*^MUT^ and *ARID1A*^WT^ cells by plotting coefficient of drug interaction (CDI) and cell fraction affected (FA) at the dosage of BRD4i: 0.1µM and ATRi 1µM. *ARID1A*^MUT^ cells were circled in blue. CDI<1 indicates synergy and CDI<0.7 indicates significant synergy. (E-F) Cell viability detection of BETi-WEE1i combination at the indicated doses in *ARID1A*^MUT^ (E) and *ARID1A*^WT^ (F) cell lines (n = 3–6; mean ± SD) 5 days post treatment. (G) CDI and FA were plotted for BRD4i-WEE1i combination. *ARID1A*^MUT^ cells were circled in blue. (H) Colony formation analysis of BRD4i-ATRi combination (left) and BRD4i-WEE1i combination (right) after 14 days drugs treatment. TOV21G, JHOC-9, OVISE, ES-2, OVKATE, and OVCAR-8 cells: ATRi (Ai) 0.1 μM + BRD4i 0.05 μM or WEE1i (Wi) 0.1 μM + BRD4i (Bi) 0.05 μM; OVMANA and OVTOKO: ATRi 0.1 μM + BRD4i 0.01 μM or WEE1i 0.1 μM + BRD4i 0.01 μM; WO-24: ATRi 0.1 μM + BRD4i 0.005 μM or WEE1i 0.1 μM + BRD4i 0.005 μM. (I) Quantification of CFA with ImageJ and the mean value were used to calculate CDI and FA.

BRD4i, ATRi, and WEE1i were selected based on monotherapy activity found in drug screen to evaluate combination strategies for CCOCs, to further improve therapeutic activity and potentially allow lower dosing strategies to minimize toxicity. BRD4i, was selected as the backbone for combination studies given its strongest monotherapy activity and selectivity for ARID1A mutant cells. To test if combination BRD4i with either ATRi or WEE1i, were more effective compared to monotherapy and if combination therapy efficacy was dependent on *ARID1A* mutation status, we evaluated BRD4i combinations using a large panel of *ARID1A*^MUT^ or *ARID1A*^WT^ gynecologic cancer lines (CCOC lines: OVTOKO, TOV21G, OVISE, JHOC-9, OVMANA, ES-2, JHOC-5, OV207, and OVCA429; HGSOC lines: WO-24, OVKATE, and OVCAR-8; Fig. 1). Consistent with their *ARID1A* mutation status, ARID1A protein is undetectable in *ARID1A*^MUT^ cell lines (OVTOKO, TOV21G, OVISE, WO-24, JHOC-9, and OVMANA), and is present in *ARID1A*^WT^ lines (ES-2, OVKATE, OVCAR-8, JHOC-5, OV207, and OVCA429) (Fig. 1A). Next, when testing different doses of BRD4i and DDRi in combination (Fig. 1B–H, Supplementary Fig. 1C). The combination of BRD4 and ATR inhibition robustly inhibited growth of *ARID1A*^MUT^ cells, especially at the low dose of BRD4i (0.1 µM) with ATRi (1 µM; highlighted in pink). This combination had limited effects on *ARID1A*^WT^ cells (Fig. 1B, C). We further calculated the coefficient of drug interaction (CDI)^46^ and fraction of cells affected (FA) after low dose BRD4i with ATRi (0.1uM, 1uM, respectively; Fig. 1D). CDI of <1.0 indicates synergy and <0.7 indicates significant synergy. BRD4i-ATRi treatment affected *ARID1A*^MUT^ lines (higher level of FA cells) with significant drug synergy (lower value of CDI) in comparison with *ARID1A*^WT^ lines (Fig. 1D; Supplementary Fig. 1C). In addition, BRD4i in combination with WEE1i, also showed significant combinatory growth inhibition in *ARID1A*^MUT^ but not *ARID1A*^WT^ lines, especially at the low dose of BRD4i (0.1 µM) with WEE1i (0.25 µ M; highlighted in pink) (Fig. 1E–F). Similar to BRD4i-ATRi, combination BRD4i-WEE1i treatment more effectively decreased viability and was synergistic in *ARID1A*^MUT^ cells (Fig. 1G; Supplementary Fig. 1). We further confirmed drug activity by evaluating effects on colony formation ability. We found that both BRD4i-ATRi and BRD4i-WEE1i combinations have significantly stronger inhibition of colony formation at very low concentrations (0.1 µM each or less) compared to monotherapies, with the degree of suppression correlating with *ARID1A* mutation status (Fig. 1H, I, Supplementary Fig. 2A). In summary, both combinations are synergistic in *ARID1A*^MUT^ but not wild type cells (Fig. 1I). Collectively, combination of BRD4i with either DDRi, ATRi or WEE1i, exhibited stronger anti-cancer effects *in vitro* than monotherapies.

### *ARID1A* loss sensitizes and overexpression decreases response to BRD4 and ATR/WEE1 inhibition

**Figure 2.**
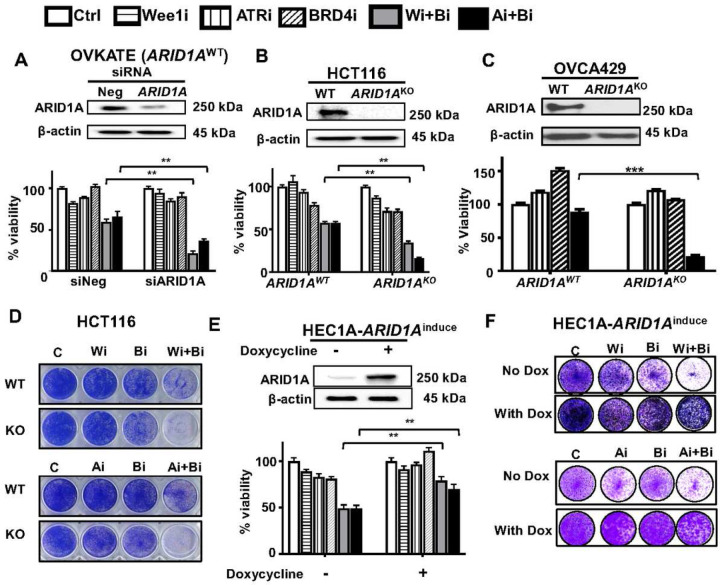
Loss of ARID1A sensitizes BRD4i-DDRi combinations and ARID1A restoration decreases the sensitivity. (A) Cell viability detection of BRD4i-ATRi and BRD4i-WEE1i combinations or monotherapy in OVKATE cells with/without ARID1A knockdown. OVKATE cells were transfected with Negative control siRNA (siNeg) or ARID1A siRNA for 24 hours and then treated with drugs for 5 days, WEE1i (0.25 µM), ATRi (0.5 µM), BRD4i (0.5 µM) monotherapy or combinations (n=3, mean+SEM). ARID1A knocking down efficacy were measured 24hours post siRNAs transfection by Western blot. (B) Cell viability detection of drugs combination in HCT116 WT and paired *ARID1A*^KO^ cells. Cells were treated with WEE1i (0.25 µM), ATRi (0.5 µM), BRD4i (0.1 µM) monotherapy or combination for 5 days (n=3, mean+SEM). ARID1A protein expression was measured by western blot in both cells. (C) Inhibitory efficacy of BRD4i-ATRi was tested in OVCA429 and paired OVCA429 *ARID1A*^KO^ cells. The cells were treated with ATRi (0.5 µM), BRD4i (0.1 µM) or combination for 5 days. The ARID1A protein was detected by western blot. (D) Colony formation analysis of BRD4i-WEE1i and BRD4i-ATRi combinations in HCT116 WT and paired *ARID1A*^KO^ cells. WEE1i 0.25 μM, BRD4i 0.1 μM for BRD4i-WEE1i combination or ATRi 0.5 μM, BRD4i 0.05 μM for BRD4i-ATRi combination. (E) Detection of drugs combination by MTT assay in HEC1A ARID1A inducible cells with/without ARID1A induction. Cells with or without 1 µg/ml doxycyclin treatment for 2 days were measured for ARID1A level by western blot. WEE1i (0.1 µM), ATRi (0.1 µM), BRD4i (0.1 µM). (n=3, mean+SEM). (F) Colony formation of HEC1A ARID1A inducible cells with/without ARID1A protein induction after drugs treatments for 10 days. WEE1i 0.2 μM, BRD4i 0.05 μM for BRD4i-WEE1i, ATRi 0.2 μM, BRD4i 0.02 μM for BRD4i-ATRi.

To evaluate whether BRD4i with DDRi combination therapeutic efficacy is dependent on *ARID1A* status, we transfected *ARID1A*^WT^ OVKATE cells with ARID1A siRNA, and show downregulation of ARID1A sensitizes OVKATE cells to both BRD4i-ATRi and BRD4i-WEE1i treatments (Fig. 2A) (Both P < 0.01, siNeg vs. siARID1A, either with BRD4i-ATRi or BRD4i-WEE1i combination). Knockdown efficiency was demonstrated showing decreased ARID1A protein (Fig. 2A). Similarly, in HCT116 (colon cancer cell) and OVCA429 (CCOC) cells, two additional *ARID1A*^WT^ lines, loss of ARID1A protein by *ARID1A* CRISPR knockout led to increased sensitivity to BRD4i-DDRi combinations (Fig. 2B–C) (P < 0.01, P < 0.001 respectively). Further, colony formation assay revealed that HCT116 *ARID1A*^KO^ cells were more vulnerable to BRD4i-DDRi combinations compared to parental (Fig. 2D, Supplementary Fig. 2B). Conversely, when ARID1A expression was restored in HEC-1A (endometrial cancer cell) using a doxycycline *ARID1A*-inducible (*ARID1A*^Induce^) system, the cells were significantly less sensitive to both BRD4i-ATRi and BRD4i-WEE1i treatment as assessed by cell viability (Both P < 0.01, + doxycycline vs. - doxycycline) and colony formation ability (Both P < 0.001 + doxycycline vs. - doxycycline Fig. 2E–F, Supplementary Fig. 2C). These data indicate that loss of ARID1A sensitizes not only CCOC but also endometrial, colon and HGSOC cells to BRD4i-DDRi treatment.

### BRD4i-ATRi combination therapy is more effective than monotherapy in *ARID1A*^MUT^ compared to *ARID1A*^WT^ CCOC PDX models

**Figure 3.**
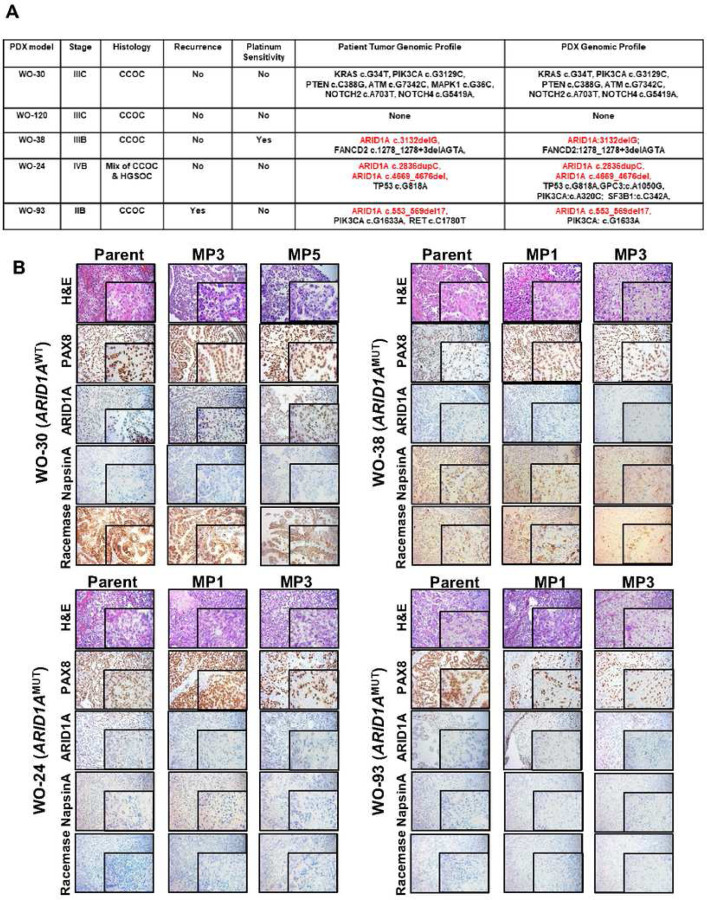
Characterization of clear cell ovarian cancer PDX models. (A) Clinical and genomic profile of clear cell ovarian cancer patient tumor tissues and PDX models. (B) Immunohistochemistry analysis of primary patient tumors, PDXs at different mouse passages (MP) were stained with anti-PAX8, anti-ARID1A, anti-NapsinA, or anti-Racemase antibody. H&E as presented to show tumor cells morphology. Images with 200X amplification and 400X (inlay) were shown.

**Figure 4.**
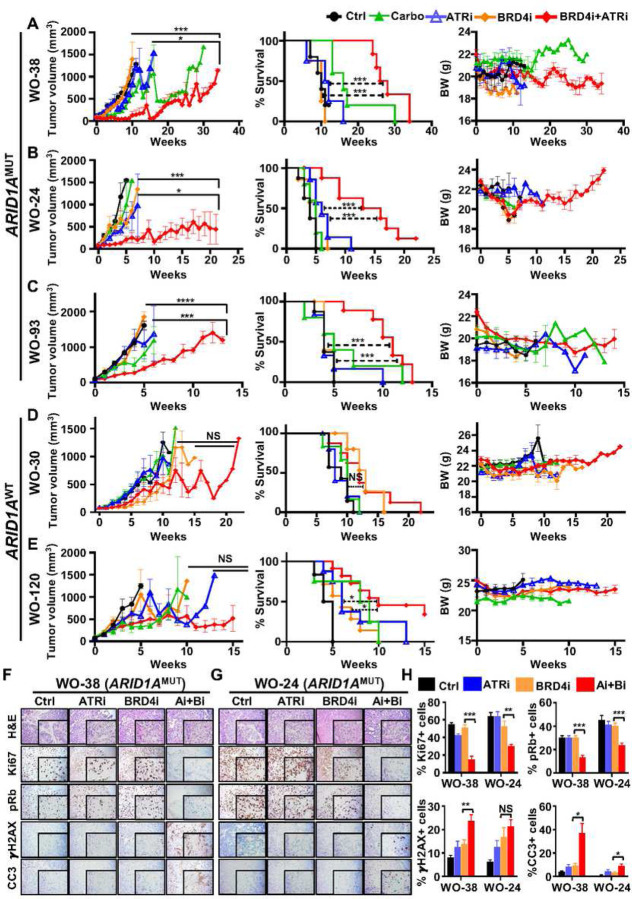
BRD4i-ATRi combination therapy is more effective than monotherapy alone in *ARID1A* mutated PDX models, but not in an *ARID1A* wild type models. (A-E) Tumor growth rate and survival curve were presented to compare BRD4i-ATRi combination with monotherapy in *ARID1A*^WT^ CCOC WO-38 (A), WO-24 (B), WO-93 (C) and *ARID1A*^MUT^ CCOC WO-30 (D), WO-120 (E). Mice were randomized to eight treatment groups once tumor volume reached 60 to 100 mm^3^. For treatment dosages: Control (vehicle), carboplatin (20 mg/kg for WO-38, WO-30, WO-120 and 30 mg/kg for WO-24, WO-93, weekly by intraperitoneal injection), ATRi (AZD6738 40 mg/kg, 5 days per week by oral gavage), BRD4i (AZD5153 1.0–1.5 mg/kg × 5 days per week by oral gavage except WO-24 and WO-93 for 7 days per week), ATRi + BRD4i (AZD5153 1.0–1.5 mg/kg × 5 days per week by oral gavage except WO-24 and WO-93 for 7 days per week; AZD6738 40 mg/kg × 5 days per week except WO-30 for 30 mg/kg). (F-H) H&E staining and immunohistochemistry detection of Ki67, p-Rb, gH2AX, and cleaved caspase 3 in WO-38 (F) and WO-24 (G) PDX tissues. The percentage of positive cells were quantification by image J (H).

There has previously been a lack of well-characterized *in vivo* experimental models for CCOC. We and other groups have shown that patient-derived xenograft (PDX) models represent the architecture and genomics of the original patient tumor, demonstrating the natural progression of ovarian cancer, and mimicking the drug response of the patient^47–53^. In this study, we developed 8 PDX models from CCOC patients using an orthotopic transplant technique (suture tumor chunk to the ovary) in NSG mice which results in primary ovarian tumors with metastasis to the peritoneal cavity similar to what is observed in patients with advanced disease. We characterized these 7 CCOC PDX models and genomic profiles are shown (Fig. 3A: WO-30, WO-120, WO-38, WO-24, and WO-93; Supplementary Tab. 1; WO-28, and WO-36). Among 8 CCOCs, three *ARID1A*^MUT^ models (WO-38, WO-24, and WO-93) and two *ARID1A*^WT^ PDX (WO-30 and WO-120) were used to evaluate BRD4i-DDRi combinations (Fig. 4). ARID1A protein expression status was confirmed by immunohistochemistry showing loss of ARID1A in WO-38, WO-24, and WO-93 and retention of ARID1A in WO-30 (Fig. 3B). PAX8, an ovarian carcinoma biomarker^54^, is highly expressed in all models (Fig. 3B). Napsin A and racemase are two proteins frequently expressed in clear cell carcinomas of the gynecological tract^55,56^. WO-38 showed high expression of both proteins. WO-30 has high levels of racemase expression, and WO-24 and WO-93 models, show minimal protein expression. Further H&E staining identified WO-30, WO-38, and WO-93 as CCOC and WO-24 as a mixture of CCOC and HGSOC (Fig. 3B). The morphology and protein expression panel of all patients’ tumor samples and corresponding PDXs at mouse passage 1 (MP1), MP3, and MP5 maintained.

In WO-38 (*ARID1A*^MUT^) and WO-24 (*ARID1A*^MUT^), although minimal tumor growth suppression was observed with monotherapies (carboplatin, WEE1i, ATRi, and BRD4i), the BRD4i-ATRi combination led to significant decreases in tumor volume (WO-38: ATRi vs. combo, P = 0.021, BRD4i vs. combo P = 0.005; WO-24 ATRi vs. combo, P = 0.016, BRD4i vs. combo P = 0.0003) (Fig. 4A–B, Supplementary Fig. 1A–B, Supplementary Table 2). Prolongation of overall survival was significant with BRD4i-ATRi but not BRD4i-WEE1i treatments (BRD4i-ATRi: WO-38: ATRi vs. combo, P= 0.001, BRD4i vs. combo P = 0.001; WO-24 ATRi vs. combo, P= 0.003, BRD4i vs. comb P= 0.002; BRD4i-WEE1i P>0.05 for WEE1i vs. BRD4i-WEE1i) (Fig. 4A–B, Supplementary Fig. 3A–B, Supplementary Tab. 2). In WO-93, a very aggressive CCOC PDX model derived from a platinum-resistant recurrent tumor, BRD4i-ATRi treatment demonstrated a statistically significant increase in survival (ATRi vs. combo, P= 0.0015, BRD4i vs. combo P < 0.001) and antitumor effect that was maintained for > 10 weeks compared to control or monotherapies (ATRi vs. combo, P< 0.001, BRD4i vs. comb P < 0.001; Fig. 4C, Supplementary Table 2). On the other hand, in this model and the other *ARID1A*^MUT^ PDX models, combination BRD4i-WEE1i was not very effective nor superior to monotherapy arms (Supplementary Fig. 3C, Supplementary Tab. 2).

Importantly, in *ARID1A*^WT^ PDX models, WO-30 and WO-120, BRD4i-ATRi treatment did lead to tumor suppression but effects were less durable and survival benefit less apparent compared to that in *ARID1A*^MUT^ models. Finally, anti-tumor and survival benefits were minimal with the BRD4i-WEE1i combination compared to monotherapy in *ARID1A*^WT^ PDXs (Fig. 4D–E, Supplementary Fig. 3D–E, Supplementary Tab. 2). The drug doses used either as monotherapy or in the BRD4i-DDRi combinations were tolerable in mice with minimal effects in decreasing mice body weight (Fig. 4A–E, Supplementary Fig. 3A–E). Collectively, these findings indicate that the combination BRD4i with ATR inhibition, promotes significant tumor regression and increased survival in *ARID1A*^MUT^ tumors, compared to *ARID1A*^WT^ models. When comparing the two DDRi in combination with BRD4i, the BRD4i-ATRi combination exhibited more robust and durable anti-tumor activity and better improvement in overall survival compared to BRD4i-WEE1i.

Drug effects on markers of proliferation, cell cycle regulation, DNA DSB, and apoptosis were evaluated in PDX tumor tissues. The BRD4i-ATRi combination significantly decreased Ki67 (P<0.001 and <0.01), pRb (P<0.001), and increased γH2AX (P<0.01 and P>0.05) and cleaved caspase 3 (P<0.05) when compared to BRD4i monotherapy, respectively, in WO-38 and WO-24 in ARID1A^MUT^ PDXs (Fig. 4F–H; (Supplementary Fig. 3F–G). Similar trend was seen in *ARID1A*^WT^ PDX models but less apparent (Supplementary Fig. 3F). These preclinical PDX trial results suggest that BRD4i-ATRi combination is a new tolerable and active therapeutic option that is superior to standard chemotherapy (carboplatin) and BRD4i-WEE1i in *ARID1A*^MUT^ CCOCs, warranting further development.

### Combination BRD4i-ATRi affects cell cycle regulation in *ARID1A* mutant cells

**Figure 5.**
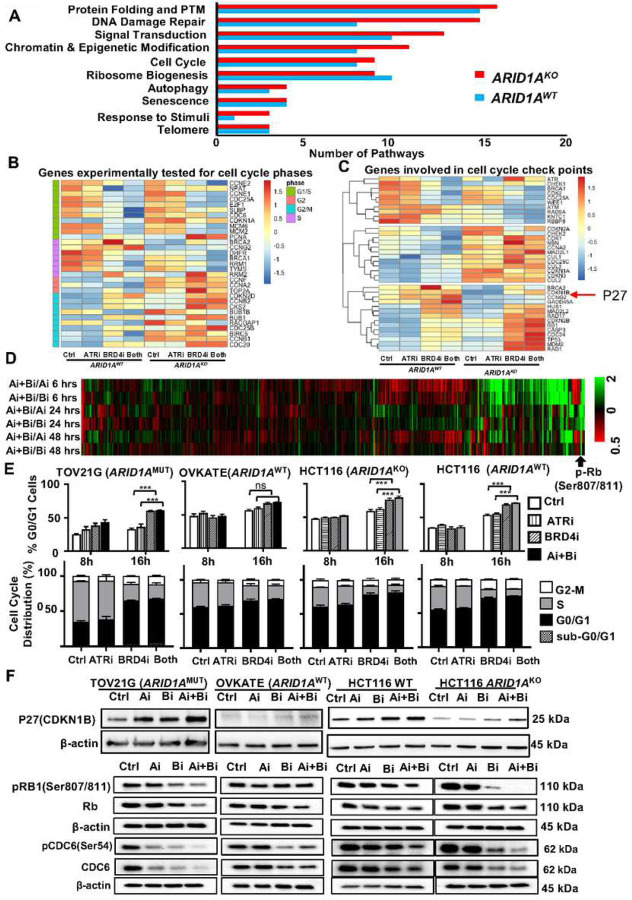
BRD4i-ATRi combination induced G1 cell cycle arrest in cells with *ARID1A* loss. (A) Analysis of pathways impacted by BRD4i-ATRi treatment in HCT116 WT and HCT116 *ARID1A*^KO^ cells. HCT116 WT and HCT116 *ARID1A*^KO^ cells were treated with BRD4i 0.1 µM, ATRi 0.1 µM or combination for 12 hours and the transcriptome was detected by RNA sequence. Data was analyzed and heatmap was generated in R software. (B) Analysis of cell cycle phases related genes expression alteration by BRD4i-ATRi treatment. (C) Analysis of the genes involved in cell cycle checkpoints affected by BRD4i-ATRi treatment. Red arrow points at the p27, which is upregulated by BRD4i-ATRi in HCT116 *ARID1A*^KO^ cells. (D) Proteomic analysis of HCT116 WT and HCT116 *ARID1A*^KO^ cells by RPPA. The cells are treated with Control, ATRi 0.1 µM (Ai), BRD4i 0.1 µM (Bi), or combination for 6, 24, 48 hours. In the RPPA analysis, the samples were probed with 445 antibodies to show the relative expression level of the proteins. The quantified data was normalized by each Control, and Ai+Bi/Ai and Ai+Bi/Bi are calculated by each time point. The ratios of HCT116 WT/HCT116 *ARID1A*^KO^ are made and the data are sorted by Ai+Bi/Ai for 24 hours. Heatmap is created by Closter 3.0 and TreeView3 software. The heatmaps show change in cell cycle-related genes. Black arrow points to pRB expression, which is significantly decreased by BRD4i-ATRi combination in HCT116 *ARID1A*^KO^ cells comparing to HCT116 WT cells. (E) Cell cycle analysis of drugs effect in TOV21G (*ARID1A*^MUT^), OVKATE (*ARID1A*^WT^), HCT116 *ARID1A*^KO^, and HCT116 WT cells. Cells were treated with Control, ATRi 0.1 µM, BRD4i 0.1 µM or combination for 8 or 16 hours. Cells were pretreated with BrdU (10 µM) treatment for 2 hours before collection. % G0/G1 cells (Upper) at 8 or 16 hours and Cell cycle distribution (Lower) at 16 hours post treatment were presented. Data was shown as mean+SEM. (F) Detection of p27 (Upper), p-Rb (middle), and p-Cdc6 (Bottom) in indicated cells by western blot. Cells were treated with ATRi 0.1 µM (Ai), BRD4i 0.1 µM (Bi) or combination for 24 hours in samples for p27 and p-Rb detection. For p-Cdc6 detection, cells were treated with Control, ATRi 0.5 µM (Ai), BRD4i 0.5 µM (Bi), or ATRi 0.5 µM + BRD4i 0.5 µM (Ai+Bi) for 24 hours. β-actin was used as internal control. Total Rb protein and total Cdc6 protein were included as control.

To investigate the underlying molecular mechanism of synergy of BRD4i combinations in the *ARID1A* loss setting, we performed RNA-seq and reverse phase protein array (RPPA) analyses after treatment with BRD4i, ATRi or combination in HCT116 WT (*ARID1A*^WT^) and HCT116 KO (*ARID1A*^KO^) cells (Fig. 5A–D). We focused our mechanistic studies on the BRD4-ATRi combination given its improved activity compared to BRD4i-WEE1i. RNA-seq analysis showed that BRD4i-ATRi treatment of *ARID1A*^KO^ cells leads to altered gene expression in several pathways including protein folding, DNA damage repair, signal transduction, chromatin and epigenetic modulation and cell cycle compared to wild type cells (fold change > 2, FDR < 0.1; BRD4i-ATRi vs. control for *ARID1A*^MUT^ vs. wildtype; Fig. 5A). In further analysis of cell cycle phase related genes, although the ATRi treatment impact on genes expressed in the S-phase of cell cycle were subtle, BRD4i treatment resulted in a significant downregulation, and the effect was further enhanced by combination of BRD4i-ATRi treatment (Fig. 5B). Furthermore, the repression was more evident in HCT116 cells carrying *ARID1A*^KO^ mutation, suggesting that ARID1A plays a key role in attenuating efficacy of the combination. In addition to repression of genes expressed in S-phase, several genes involved in G1/S progression were also affected. Specifically, CDK2 and CDC25A were down-regulated while CDKN1B, CDKN2B, and RB1, negative regulators of G1/S progression, were upregulated (Fig 5C). This pattern was more pronounced with BRD4i-ATRi combination treatment than with BRD4i monotherapy, and ATRi alone only had subtle impact, potentially explaining the synergistic effect of the combination therapy. Further, RPPA analyses showed that p-Rb protein level was decreased in HCT116 *ARID1A*^KO^ compared to that of *ARID1A*^WT^ after treatment with BRD4i-ATRi combination (Fig. 5D), regardless of the treatment duration (6, 24, or 48 hours), further suggesting the inhibition of cell cycle progression with drug treatments. To further evaluate cell cycle effects after BRD4i-ATRi treatment, we tested the cell cycle distribution by flow cytometry. Consistent with the RNA-seq results, flow cytometry analysis demonstrated reduction of the S-phase cell population after the drug treatments (Fig. 5E, Supplementary Fig. 4). Specifically, when cells were treated with monotherapies or low-dose combinations (0.1 µM of each drug), BRD4i monotherapy and DDRi-BRD4i combination (WEE1i-BRD4i or ATRi-BRD4i), a robust G1 cell cycle arrest was observed in TOV21G (*ARID1A*^MUT^) compared to OVKATE (*ARID1A*^WT^) cells, and this effect was more enhanced in HCT116 *ARID1A*^KO^ compared with the isogenic matched HCT116 *ARID1A*^*WT*^ cells (Fig. 5E). Further, Western blot analysis showed that the protein levels of p-Rb in TOV21G (*ARID1A*^MUT^) and HCT116 *ARID1A*^KO^ were significantly decreased compared to that in OVKATE (*ARID1A*^WT^) and HCT116 *ARID1A*^WT^ cells, suggesting G1 arrest in ARID1A deficient cells (Fig. 5F). Thus, although DDRi and BRD4i treatments have strong effects on cell cycle progression, *ARID1A*-loss led to higher susceptibility to BRD4i-associated G1 cell cycle arrest. We also examined levels of protein expression of additional cell cycle regulation factors after treatment with ATRi and BRD4i. ATR and BRD4 downstream proteins, pChk1, CtIP, and c-Myc, were downregulated by BRD4i-ATRi independent of ARID1A protein level (Supplementary Fig. 5). The protein level of P27 (CDKN1B), an inhibitor of cell cycle progression, was increased after drug treatment (Fig. 5F), similar to its RNA level change observed with RNAseq. Cdc6, cell division cycle 6, is a critical regulator of DNA replication and required for recruiting minichromosome maintenance (MCM) protein complexes to DNA. Cdc6, a key regulator of cell cycle, also works as a cycle checkpoint maintenance which orchestrates S phase and mitosis^57^. BRD4i-ATRi treatment led to significant decreases of p-Cdc6 and total Cdc6 in cell lines carrying *ARID1A*^MUT^ or *ARID1A*^KO^ (Fig. 5F), again suggesting inhibition of cell cycle progression was more pronounced in the setting of ARID1A loss.

### BRD4i-ATRi combination decreases homologous recombination and induces cell apoptosis in CCOC with ARID1A loss

**Figure 6.**
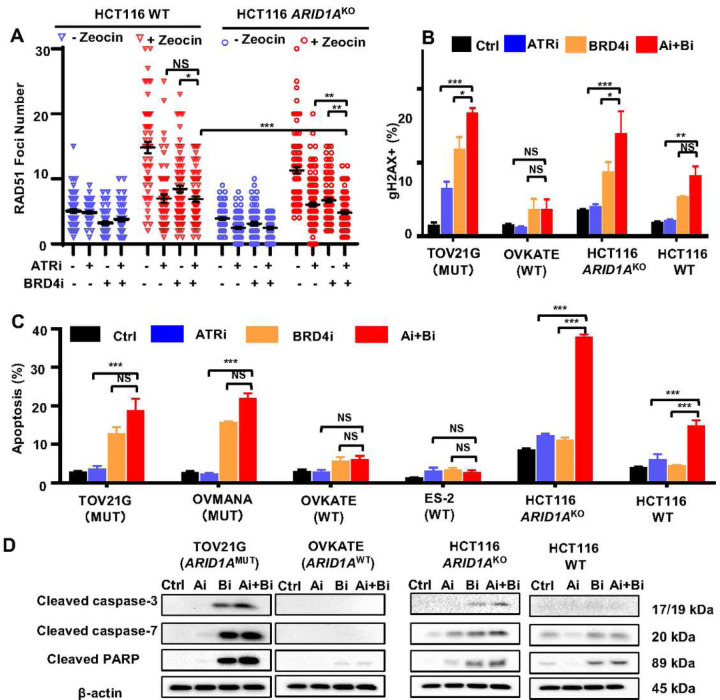
BRD4i-ATRi combination therapy reduced homologous recombination, induced DNA damage, and led to cell apoptosis in *ARID1A* mutant CCOC. (A) Measurement of Homologous recombination by detecting RAD51 foci formation. HCT116 WT and HCT116 *ARID1A*^KO^ cells were treated with 0.1 µM BRD4i and 1 µM ATRi for 6 hours. Zeocin was added during 6 hours drug treatment. Immunofluorescence staining of RAD51 and Gemini was performed. RAD51 foci number in Gemini positive cells were counted and plotted. Individual data points and median ± SEM were shown. (B) Detection of gH2AX positive cells by flow cytometry. TOV21G (*ARID1A*^MUT^), OVKATE (*ARID1A*^WT^), HCT116 *ARID1A*^KO^, or HCT116 WT cells were treated with Control, ATRi (0.25 µM), BRD4i (0.25 µM) or combination for 24 hours before gH2AX (Ser139) staining. Data are shown as mean+SEM. (C) Detection of cell apoptosis in *ARID1A* mutant (TOV21G and OVMANA), *ARID1A* WT (ES-2 and OVKATE) and HCT116 *ARID1A*^KO^, HCT116 WT cells. TOV21G and OVMANA, ES-2 and OVKATE cells were treated with 0.1 µM ATRi, 0.1 µM BRD4i or combination for 5 days. HCT116 *ARID1A*^KO^ and HCT116 WT were treated with ATRi (0.5 µM), BRD4i (0.1 µM) or combination for 5 days. Graph bars show mean+SEM. (D) Western blot detection of apoptotic protein markers in *ARID1A* mutant and WT cells. The cells were treated with ATRi (Ai, 0.5 µM), BRD4i (Bi, 0.5 µM) or combination for 24 hours.

BRD4i treatment decreased and BRD4i-ATRi further decreased expression of homologous recombination (HR) related genes, such as *BRCA1* in ARID1A KO cells by RNAseq analysis (Fig. 5B–C). To examine whether BRD4i-ATRi decrease HR, we evaluated RAD51 foci (a marker for HR), by immunofluorescence staining, in geminin (a marker for cells in late S and G2 phase) positive cells. Cells had minimal RAD51 foci at baseline (Blue symbols, Fig. 6A). By inducing DNA breaks with zeocine treatment, RAD51 foci dramatically formed in HCT116 WT and KO cells. BRD4i and ATRi monotherapy each decreased RAD51 foci. The BRD4i-ATRi combination treatment further reduced RAD51 foci in *ARID1A* KO cells but less in WT cells compared to monotherapy (Fig. 5A, BRD4i vs. BRD4i-ATRi in WT: P>0.05, BRD4i vs. BRD4i-ATRi in KO: P<0.01, WT BRD4i-ATRi vs. KO BRD4i-ATRi: P<0.001). We hypothesized that DNA repair function in *ARID1A*^KO^ cells treated with BRD4-ATRi would be further inhibited resulting in increased DNA DSB. After treating cells with BRD4-WEE1i or BRD4i-ATRi, dramatic increases of γH2AX (a marker for DNA DSB) positive cells were observed in cell lines carrying *ARID1A*^MUT^ (TOV21G) or *ARID1A*^KO^ alleles (HCT116) compared with *ARID1A*^WT^ (Fig. 6B, Supplementary Fig. 6). Given that drug effects on cell cycle and DNA repair was likely dependent on *ARID1A* mutation status, we next evaluated whether DNA damage led to apoptosis. Combination inhibition with BRD4-ATR significantly induced cell apoptosis in *ARID1A* mutant cells (TOV21G, OVMANA) and *ARID1A*^KO^ cells (HCT116 *ARID1A*^KO^, OVCA429 *ARID1A*^KO^) but minimal apoptosis in *ARID1A*^WT^ cells (OVKATE, ES2, HCT116, OVCA429) by Anexin V/PI staining (Fig. 6C, Supplementary Fig. 7). We then further tested levels of apoptotic protein markers (cleaved caspase-3, cleaved caspase-7, and cleaved PARP) in TOV21G and HCT116 *ARID1A* deficient cells, and OVKATE and HCT116 (*ARID1A*^*WT*^) cells after treating with ATRi, BRD4i, or the combination. Apoptosis marker proteins were increased more by BRD4i-ATRi in cells with ARID1A loss compared to *ARID1A*^WT^ cells (Fig. 6D).

**Figure 7.**
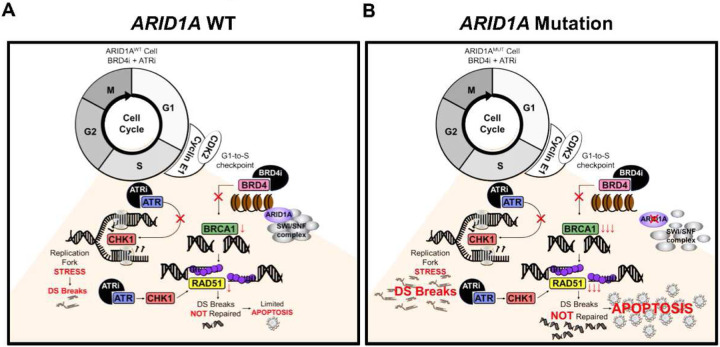
Schematical diagram of BRD4i-ATRi mechanism of function. (A) In ARID1A wild type CCOC cells, BRD4i has minimal effect in decreasing BRCA1 expression and RAD51 loading, ATRi has limited effect in inducing replication stress. These lead to minimal effect of BRD4i-ATRi combination in inducing cell apoptosis. (B) In *ARID1A* mutant CCOC, inhibition of BRD4 decreases BRCA1 transcription, prevents RAD51 loading, leading to lack of homologous recombination and increasing DNA double strand (DS) break. ATRi inhibits CHK1 and activates CDK1 activation, resulting in loss of G2/M checkpoint, and also ATRi increased replication stress and DS break. Combination of BRD4i-ATRi significantly induced DNA damage and cell apoptosis.

Taken together, DDRi-BRD4i combination treatment significantly activated DNA damage and apoptosis pathways more so in cells with *ARID1A*^MUT^ or *ARID1A*^KO^ compared to *ARID1A*^WT^. In CCOC with ARID1A loss, inhibition of BRD4 decreases BRCA1expression levels and prevents RAD51 loading, thus leading to decreased homologous recombination and increased DNA double strand (DS) DNA breaks. ATRi inhibits CHK1 and activates CDK1 activation, resulting in loss of the G2 checkpoint, at the same time, it also increases replication stress and DS DNA breaks (Fig 7). Combination of BRD4i-ATRi significantly induced DNA damage and cell apoptosis (Fig. 7). However, in ARID1A wild type CCOC cells, BRD4i has less effect in decreasing BRCA1 expression and RAD51 loading, and leads to minimal induction of DNA damage and cell apoptosis when in combination with ATRi (Fig. 7).

## Discussion

Clear cell ovarian cancer (CCOC) is one of the most challenging subtypes of ovarian cancers to treat. They are intrinsically resistant to standard chemotherapy and thus, developing effective treatment for this subtype is critical and an unmet medical need ^3–5^. The *ARID1A* gene, a member of the SWI/SNF family, is the most prevalently mutated gene in CCOC^7^. Functions of ARID1A include modulating chromatin structures and gene transcription, as well as directly facilitating DNA accessibility to DNA damage repair proteins (e.g. p53, BRCA1)^17^. Prior studies have also shown that loss of ARID1A impairs G2/M checkpoint initiation, increasing reliance on the DDR^36,37^. Thus, we hypothesized that pathogenic mutations in CCOC, *ARID1A* mutations in particular, could be exploited by targeting DNA damage response (DDR) and chromatin regulation.

To identify effective drugs for CCOC treatment, either as a monotherapy or combination therapy, we performed a drug screen with various targeted drug candidates including standard chemotherapy, epigenetic regulators, and inhibitors of tyrosine kinase signaling pathways and the DNA damage response. We identified BRD4i followed by DDR inhibitors (ATRi, WEE1i) as the most effective drugs in CCOC cells and cells with *ARID1A* mutations were especially sensitive to BRD4i (Supplementary Tab. 1). Consistent with these observations, *ARID1A* loss is synthetically lethal with ATR inhibition in large-scale genetic screen^37^. Additionally, *ARID1A* mutations sensitize most ovarian clear cell carcinomas to BET inhibitors identified through a kinome lethality screen^34^.

Given the rapid emergence of resistance to monotherapy in cancer, we investigated whether combination BRD4i with WEE1 or ATR inhibition would be a strategy to exploit *ARID1A* loss by targeting two critical pathways critical for survival. To validate drug screening results, a large library of *ARID1A* mutant and wild type CCOC cells were tested with BRD4i in combination with ATRi or with WEE1i. We found both combinations were synergistic by coefficient of drug interaction (CDI) scores (CDI <1=synergism)^46^ in decreasing viability and colony formation more so in the *ARID1A*^MUT^ lines compared to *ARID1A*^WT^ at *in vitro* doses lower or comparable to doses used in the clinic (BRD4 10–100nM, WEE1i 100–250nM, ATRi 100–1000nM; Fig. 1). We validated that response to BRD4i with ATRi or BRD4i with WEE1i was dependent on *ARID1A* loss status by *ARID1A* knockout or knock down increasing response to the combination; and overexpression of ARID1A, decreasing the response to the BRD4i-DDRi combination. Others have shown that combination inhibition of BRD4 and ATR demonstrates synergistic cytotoxic activity in other cancers such as lymphomas, melanoma and high-grade serous ovarian cancer ^58–60^, however *ARID1A* loss as a biomarker of sensitivity was not explored. We further show that drug sensitivity was impaired by increased cellular levels of ARID1A protein not only in CCOC, but in endometrial, colon, and HGSOC cells (Fig. 2) warranting further investigation of this combination in other cancer types with *ARID1A* loss. Eric: Collectively, these results support the concept that *ARID1A* pathogenic variants sensitize cancer cells to the BRD4i-ATRi combination and may serve as biomarkers to select patients for this efficacious treatment.

Advancements in CCOC treatments have been hampered by lack of preclinical models. PDX models developed represented similar genomic (e.g., *ARID1A, PIK3CA, KRAS* etc.) and protein (PAX8, ARID1A, napsin A, racemase) profiles to the native patient tumor from which they were derived supporting use of these models as surrogates of the patient tumor. Using the CCOC PDX platform, we compared BRD4i-ATRi and BRD4i-WEE1i vs. monotherapy and carboplatin in *ARID1A*^MUT^ (WO-38, WO-24, and WO-93) and wild type models (WO-30, WO-120; Fig. 4 and Supplementary Fig. 1). Although some tumor growth suppression was observed with monotherapies (e.g carboplatin, ATRi, WEE1i and BRD4i), BRD4i-ATRi combination was tolerable and led to significant robust decreases in tumor volume and increased overall survival relative to single-agent therapies that were maintained for >15–35 weeks in *ARID1A*^MUT^ in contrast to modest effects in the *ARID1A*^WT^ PDX models (Fig. 4). Indeed, in the *ARID1A*^WT^ (WO-30, WO-120) PDXs, BRD4i-DDRi did not demonstrate significant antitumor activity (Fig. 4D, E). BRD4i-DDRi combinations were tolerated at clinically relevant doses as shown by stable body weight. Interestingly, the BRD4i-ATRi was more active than the BRD4i-WEE1i likely because ATR has distinct roles in protecting replication fork stability in S phase in addition to G2/M regulation, a key function of the downstream WEE1 kinase. Alternatively, the dual effects of WEE1i on G1/S and G2/M progression may compromise its cooperation with BRD4i. In summary, more relevant to clinical applications, BRD4i-ATRi treatment causes complete tumor regression in chemotherapy-resistant CCOC PDX tumors more so than monotherapy in an ARID1A level-dependent manner.

Mechanistically, RNA-seq and RPPA drug response studies revealed that cell cycle regulators of G1/S including p27 and pRB, were significantly reduced by BRD4i-ATRi combination in an ARID1A mutation-dependent manner. p27^Kip1^ arrests cells at G1 by inhibiting the activity of cyclin E-CDK2 complexes^61^, and phosphorylation of RB1 and CDC6 mark passage from G1 into S phase. ^62^. We found combination BRD4i-ATRi increased G1 arrest and decreased S phase, which indeed correlates with decreased phosphorylation of RB and CDC6 more so than monotherapy in a manner dependent on ARID1A mutation (Fig 5.). Similarly, others have shown BRD4 inhibition deregulates CDC6 activity and results in aberrant DNA replication re-initiation and sensitization to replication stress-inducing agents^58^. Since ARID1A-null or KO is defective in the G2M checkpoint^36^, it is possible that the combination of replication fork collapse and G/M checking inhibition with *ARID1A* mutation allows damaged cells to pass through M phase and arrest in G1 through an alternative checkpoint mechanism (e.g. ATM), ultimately leading to apoptosis. Similarly, ATR-mediated phosphorylation of CHK1 is higher in ARID1A-mutant or deleted cells (Supplementary Fig. 3), thus making these cells more reliant on ATR function to stabilize stalled replication forks^37^.

Given RNA seq analysis identified DNA Damage Repair pathways as significantly more effected in *ARID1A*^MUT^ compared to wildtype cells, we evaluated drug effects on homologous recombination (HR), the primary mechanism to repair DNA DSBs^63^. BRCA1 regulates RAD51 deposition in response to DNA damage by recruiting PALB2 and BRCA2^64^. BRCA2 loads RAD51 onto resected DSBs for HR ^65,66^. RAD51 foci formation has been used as a functional biomarker of HR^67^ which is critical for DNA repair. RNA-seq revealed that BRD4i decreased BRCA1 expression, with a further decrease observed following BRD4i-ATRi treatment in the *ARID1A* mutant cells (Fig. 5).

*ARID1A* loss and BRD4i increases reliance on ATR function^37,60^. Thus, we examined the effect of ATRi added to BRD4i on ɣH2AX, a DNA double stand break (DSB) marker^68^ and cleaved caspase 3, a marker for apoptosis^69^. Treatment of *ARID1A*^MUT^ PDXs indeed exhibited significantly increased ɣH2AX and CC3-positive cells compared with *ARID1A*^WT^ (Fig. 4 and Supplementary Fig. 1) suggesting ARID1A has a role in maintaining genomic stability. However, ATRi and BRD4i caused more DSBs in combination combined to monotherapies, and these breaks are further increased by *ARID1A* loss. Therefore, we observed that BRD4i-ATRi combination significantly induced more γH2AX in ARID1A deficient CCOC lines compared to wild type models.

Regarding the mechanism by which BRD4i-ATRi increase breakage, we also found that monotherapy each decreased RAD51 foci in *ARID1A*^KO^ cells, which is consistent with previous findings in other genetic contexts^49,70^. However, combination of BRD4i-ATRi decreased RAD51 foci more so than monotherapies, suggesting that this combination further inhibits HR-mediated repair of DNA double strand breaks, leading to apoptosis (Fig. 6). The mechanisms underlying these effects varied and are likely cumulative. For example, *ARID1A* loss causes decreased transcription of HR genes^18^. BETi may cooperate with *ARID1A* loss in producing these changes^71^. *ARID1A* loss leads to the ATR-mediated phosphorylation of CHK1, which implies replication fork stalling (Supplemental Fig. 5). In addition, inhibition of the ATR-CHK1 pathway has been shown to suppress the accumulation of RAD51 on DSBs^72^. Although each drug demonstrates distinct mechanisms of action, the combination of BRD4i and ATRi synergizes with *ARID1A*^MUT^ to generate DSBs and permit aberrant progression though M phase into G1, where alternative checkpoint mechanisms are triggered (Fig. 7).

In summary, our study identified a novel drug combination, BRD4i-ATRi that exploits the most common genetic vulnerability, ARID1A loss in CCOCs, which are in dire need of new treatment options. Combination low dose BRD4i-ATRi was tolerable and highly effective in aggressive platinum resistant CCOC models justifying further evaluation of this combination in the clinic.

## Online Methods

### Materials

McCoy’s 5A (#16600-082), Medium 199 (#11150-059), RPMI1640 (#11875-085), DMEM/F12 (#11320-033), 1% Penn Strep (Penicillin/Streptomycin; P/S, #15140122), MEM Non-Essential Amino Acids (NEAA) Solution (100X, #11140-050), DPBS (#14040-117), and Trypsin-EDTA (0.25%, #25200-056) were purchased from Gibco (Life Technologies, Grand Island, NY). MCDB105 (#117-500) was purchased from Cell Applications Inc. (San Diego, CA). Alpha-MEM was obtained from Irvine Scientific (Irvine, CA) OCMI-E was purchased from University of Miami (Live Tumor Culture Core at Sylvester Comprehensive Cancer Center, University of Miami Miller School of Medicine, Miami, FL). Fetal bovine serum (FBS, #F2442) and Cholera Toxin from Vibrio cholerae (#C8052) were purchased from Sigma-Aldrich (St. Louis, MO). Dimethyl Sulfoxide (DMSO, #CAS 67-68-5) was purchased from Thermo Fisher Scientific (Waltham, MA).

### Cell culture

TOV21G, OVMANA, OVTOKO, HEC-1-A, ES-2, and OVCAR-8 were obtained from the American Tissue Type Collection (Manassas, VA) and as a gift from Dr. Gottfried Konecny (ULCA, Los Angeles, CA). JHOC-5 and JHOC-9 was purchased from RIKEN BioResource Cener (Tsukuba, Ibaraki, Japan). OVISE was a gift from Dr. David Huntsman (The University of British Columbia, Vancouver, BC). OVCA429 and OVCA429 ARID1A knockout (*ARID1A*^KO^) cells were gifts from Dr. Rugang Zhang (Wistar Institute, Philadelphia, PA). OV207 was gift from Dr. Vijayalakshmi Shridhar (Mayo Clinic, Rochester, MN). WO-24 primary ovarian cancer tumor culture was generated in our laboratory. Fresh tumor obtained at the time of ovarian cancer surgery was minced, digested and grown in OCMI-E media with the addition of 30 ng/mL Cholera Toxin. The cells were grown in hypoxia (5% O2) with 5% CO2 over 20 passages and were stained for PAX 8 and cytokeratin 7 by immunofluorescence. Once established, WO-24 cells were maintained in RPMI-1640 under normoxia with 10% FBS. OVKATE was purchased from JCRB Cell Bank (Ibaraki, Osaka, Japan). HCT116 WT and HCT116 *ARID1A*^KO^ (#HD 104-049, homozygous knockout of ARID1A by knockin of premature stop codon (Q456*)) were purchased from Horizon Discovery (Waterbeach, Cambridge, UK). HEC-1-A carrying Tet-inducible ARID1A (HEC1A-ARID1A^Induce^) was established and from Dr. Tian-Li Wang lab^73^. TOV21G was cultured in 1:1 mixture of MCDB105 and Medium 199 supplemented with 15% FBS and 1% P/S. OVMANA, OVISE, OVTOKO, OVCA429, OVKATE, OVCAR-8, HEC116 WT and HCT116 *ARID1A*^KO^ were cultured in RPMI1640 supplemented with 10% FBS and 1% P/S. JHOC-9 and JHOC-5 was cultured in DMEM/F12 supplemented with 10% FBS and 0.1mM NEAA. OV207 was maintained in alpha-MEM supplemented with 20% FBS and 1% P/S. ES-2, HEC-1-A, and HEC1A-ARID1A^Induce^ were cultured in McCoy’s 5A supplemented with 10% FBS and 1% P/S. All cells were incubated at 37 °C with 5% CO2. All cell lines were authenticated using short tandem repeat (STR) profiling and tested to be free of Mycoplasma using MycoAlert (Cambrex) at the Cell Center Service at the University of Pennsylvania.

### *In vitro* cell viability assay (MTT assay)

Cells were seeded in 96-well plates based on doubling time (Supplementary Fig. 8) (5 × 10^3^ cells/well for cells with medium growth rate) and treated with BRD4i (AZD5153, AstraZeneca), ATRi (AZD6738, AstraZeneca), WEE1i (AZD1775, AstraZeneca) or combinations at indicated dosages for 5 days. For drug screen, the cells were treated with different concentration of carboplatin (APP Pharmaceuticals, LLC, Schaumburg, IL), paclitaxel (Hospira, Inc, Lake Forest, IL), olaparib (AstraZeneca, Cambridge, UK), AZD1775, AZD6738, AZD5153, JQ1 (Selleck Chemicals, Houston, TX), GSK126 (Selleck Chemicals), EPZ6438 (Selleck Chemicals), Decitabine (Selleck Chemicals), or BKM120 (Selleck Chemicals) for 5 days. 10 µl of 5 mg/mL MTT (#J19265, Affymetrix) solution were added into cells and at 37°C for 4 hours. The MTT formazan was fully dissolved with 100 µL DMSO after supernatant discarded. The absorbance was measured at OD=570 nm. The assays were performed at least in triplicate (n=3) and the data are shown as mean + SEM. Log dose response curves were constructed by GraphPad Prism 7 (GraphPad Software, San Diego, CA) and the IC_50_ (half maximal inhibitory concentration) values were calculated. To analyze the drug interaction between BRD4i-ATRi or BRD4i-WEE1i, the coefficient of drug interaction (CDI) was calculated. CDI was calculated with the following formula: CDI = AB / (A × B) where A and B are cell viability of monotherapy groups relative to control group and AB is the cell viability of the combination group relative to control group. CDI < 1 indicates synergy. CDI < 0.7 means significant synergy. CDI = 1 shows additivity. CD > 1 means antagonism^46^.

### Colony formation assay

Ten thousand of cells were plated onto 12-well plates in triplicates and treated with BRD4i (Bi) ATRi (Ai), WEE1i (Wi), or combinations for 10 or 14 days. Drugs and media were refreshed every three days. The drug dosages were Wi 0.1 µM, Ai 0.1 µM, and Bi 0.05 µM for TOV21G, JHOC-9, OVISE, ES-2, OVKATE, and OVCAR-8, Wi 0.1 µM, Ai 0.1 µM, and Bi 0.01 µM for OVMANA and OVTOKO, Wi 0.1 µM, Ai 0.1 µ M, and Bi 0.005 µM for WO-24; Wi 0.25 µM, Ai 0.5 µM, and Bi 0.1 µM for HEC116 WT and HCT116 *ARID1A*^KO^; and Wi 0.2 µM, Ai 0.2 µM, and Bi 0.02 or 0.05 µM for HEC1A-ARID1A^Induce^.. Cells were then fixed and stained with 1% paraformaldehyde, 10% methanol, and 0.05% Crystal violet in PBS for 30 minutes at room temperature. The plates were washed, scanned, and quantified by Image J (National Institutes of Health, Bethesda, MD).

### In vitro proliferation assay

Two thousand cells were seeded in 96-well plate at least in triplicate on Day 0. The cell numbers were measured by CyQUANT Cell Proliferation Assay kit (C7026, Thermo Fisher Scientific) after 24, 48, 72, 96, or 120 hours incubation following the manufacture’s protocol. Doubling times were calculated by http://doubling-time.com/.

### Western blotting

Cells were seeded into 6-well plates at 3× 10^5^ cells/well and treated with BRD4i, ATRi, WEE1i, or combinations at different times with indicated dosages. For ARID1A induction in HEC1A ARID1A^Induce^ cells, the cells were treated with 1 µg/ml of doxycyclin for 48 hours to confirm ARID1A expression, or pretreated with 1 µg/ml of doxycyclin and then drugs’ treatments. Whole cell extracts were generated by collecting adherent cells with Laemmli sample buffer (10% SDS, 20% of glycerol, and 12% of 1M Tris pH 6.8 in distilled water) and heated at 95 °C for 15 minutes. Protein concentration was measured using Pierce BCA Protein Assay Kit (#23225, ThermoFisher). Samples (30 µg protein) mixed with Laemmli SDS reducing buffer (#J61337, ThermoFisher) were loaded onto 4–15% or 7.5% Mini-PROTEAN TGX Gels (#456-1086 or #456-1026, respectively, Bio-Rad Laboratories, Inc., Hercules, CA) and separated by electrophoresis in Tris/Glycine/SDS running buffer (#1610772, Bio-Rad) with PageRuler Plus Prestained Protein Ladder (# 26619, Thermo). Proteins were transferred to PVDF membranes by Trans-Blot Turbo Transfer System (Bio-Rad) for 30 minutes or 10 minutes with different programs for medium or high molecular proteins. The membranes were blocked in 5% non-fat dry milk (#M-0841, LabScientific Inc., Highlands, NJ) in TBS (#170-6435, Bio-Rad) with 0.05% Tween-20 (#1706531, Bio-Rad) at room temperature for 30 minutes and incubated in appropriate primary antibodies diluted with 1% Bovine Serum Albumin (BSA, #A7906, Sigma-Aldrich) in TBS-T overnight at 4 °C (1:2000 for β-actin, Rb, and BRD4; 1:1000 for p-Rb, c-Myc, p-Cdc6, Cdc6, p27, Cleaved caspase-3, Cleaved caspase-7, and Cleaved PARP; and 1:500 for ARID1A, p-Chk1, and CtIP). Antibodies against ARID1A (#12354), β-actin (#3700), p-Rb (Ser807/811, #9308), Rb (#9309), p-Chk1 (Ser345, #2348), CtIP (#9201), p27 Kip1 (#2552), c-Myc (#5605), Cdc6 (#3387), p27 (#2552), Cleaved caspase-3 (Asp175, #9664), Cleaved caspase-7 (Asp198, #9491), and Cleaved PARP (Asp214, #5625) were obtained from Cell Signaling Technology (Danvers, MA, USA). Antibody against BRD4 (A301-985A100) was from Bethyl Laboratories (Montgomery, TX, USA). Antibody against p-Cdc6 (Ser54, ab75809) was from Abcam (Cambridge, MA, USA). Blots were then incubated in HRP-linked secondary antibody (Anti-rabbit IgG, #7074S and Anti-mouse IgG, #7076S, Cell Signaling) at 1:2000 dilution in blocking buffer at room temperature for 1 hour. Proteins were detected by using Clarity Western ECL Substrate (#170-5061, Bio-Rad) and imaged with ChemiDoc MP Imaging System (Bio-Rad). After initial development, membranes were stripped with Restore Western Blot Stripping Buffer (#21059, Thermo) and re-probed with antibodies to β-actin as a loading control.

### siRNA transfection

The *ARID1A*^WT^ OVKATE cells were seeded in 6-well plate at 5 x 10^5^ cells/well. The cells were transfected with 2.5 µl negative control siRNA (siNC, Non-targeting control, #D-001810-10-05, Dharmacon, Lafayette, CO) at 10 µM or siARID1A (#L-017263-00-0005, Dharmacon) using Lipofectamine RNAiMAX (# 13778150, Thermo Fisher Scientific). The cells were subjected to ARID1A protein expression detection for knockdown efficacy 48 hours post transfection or to cell viability detection with MTT assay 6 days post transfection.

### Reverse phase protein array (RPPA) analysis

HCT116 WT and HCT116 *ARID1A*^KO^ cells were treated with Control, ATRi 0.1 µM, BRD4i 0.1 µM, or combination for 6, 24, or 48 hours. 1 x 10^6^ cells were collected and sent to the RPPA Core Facility at MD Anderson Cancer Center, and RPPA analysis was performed as described previously^74^. Briefly, cell lysates were serially diluted (two-fold dilution) to 1:16 dilution, and arrayed on nitrocellulose-coated slides in an 11x11 format. Samples were probed with 445 antibodies by tyramide-based signal amplification approach and visualized by DAB colorimetric reaction. Slides were scanned on a Huron TissueScope scanner and the density was quantified by Array-Pro Analyzer. Relative protein levels for each sample were determined by interpolation of each dilution curves from the "standard curve" (SuperCurve) of the slide (antibody). SuperCurve was constructed by a script in R, and all relative protein level data points were normalized for protein loading and transformed to linear values (NormLinear). The normalized data was analyzed, and the ratio of BRD4i-ATRi/ATRi and BRD4i-ATRi/BRD4i at each time point of HCT116 WT and HCT116 *ARID1A*^KO^ cells were calculated. The value of these ratios in HCT116 WT were divided by those in HCT116 *ARID1A*^KO^ cells (HCT116 WT / HCT116 *ARID1A*^KO^) were used for heatmaps generation. Heatmap were created by Closter 3.0 and TreeView3 software.

### Cell cycle analysis

Cells were seeded on 6-well plates in triplicate at least and treated with vehicle (control), 0.1μM BRD4i, 0.1μM ATRi, 0.1μM WEE1i, or combinations for 8 or 16 hours. Cell cycle was evaluated using the FITC-BrdU Flow Kit (#559619, BD Biosciences, San Jose, CA). The cells were analyzed using a BD LSR II flow cytometer (BD Biosciences) and FlowJo (FlowJo LLC, Ashland, OR) data analysis software.

### Flow cytometry detection of γH2AX protein

TOV21G, OVKATE, HCT116 WT and HCT116 *ARID1A*^KO^ cells were seeded on 6-well plates at 3 x 10^5^ cells/well in triplicate and treated with vehicle (control), 0.25 μM BRD4i, 0.25 μM ATRi, 0.25 μM Wee1i, or combinations for 24 hours. Cells were then trypsinized, fixed with 4% paraformaldehyde for 15 minutes at room temperature, washed with PBS, and incubated with blocking buffer (1% FBS + 0.1% Triton X-100 + 0.1% Tween 20 in PBS) for 30 minutes at room temperature and then stained with γH2AX antibody (Cell Signaling Tech, 1:300 dilution in blocking buffer). The cells were washed, and incubated with secondary antibody goat anti-Rabbit IgG (H+L), Alexa Fluor^®^ 647 (#A-21245, Thermo Fisher Scientific) for 30 minutes. After removal of secondary antibodies and washing with PBS twice, the cells were then incubated with 50 μg/mL propidium iodide (#00-6990-50, Thermo Fisher Scientific) for 10 minutes and then subjected to flow cytometry analysis on a BD LSR II flow cytometer (BD Biosciences). The data were analyzed using FlowJo (FlowJo LLC.)

### Apoptosis analysis

Cells were plated on 6-well plates in triplicate and treated with vehicle (control), 0.1 μM Wee1i, 0.1 μM ATRi, 0.1 μM BRD4i, or combinations for 5 days for TOV21G, OVMANA, OVKATE, and ES-2 cell lines. HEC116 cell lines carrying WT and mutant *ARID1A* were treated with vehicle (control), 0.25 μM Wee1i, 0.5 μM ATRi, 0.1 μM BRD4i, or combinations for 5 days. OVCA429 WT and OVCA429 *ARID1A*^KO^ cells were treated with 0.1 μM BRD4i, 1 μM ATRi, or combination for 3 days. Apoptosis assay was performed with eBioscience^™^ Annexin V Apoptosis Detection Kit APC (#88-8007-74, Thermo Fisher Scientific), according to the manufacturer’s instruction. Annexin V-APC and propidium iodide (#00-6990-50, Thermo Fisher Scientific) labeled cells were detected by BD Accuri^™^ C6 Cytometer (BD Biosciences). The acquired data was analyzed with FlowJo (FlowJo LLC).

### Immunofluorescence

HCT116 WT and HCT116 *ARID1A*^KO^ cells were seeded on 24-well plates with coverslides. After 24 hours, cells were treated with ATRi (0.5 uM), BRD4i (0.1 uM), and Both for 6 hours. Zeocin (0.1mg/ml) was added during 6hr drug treatment. The cells were fixed with 4% PFA for 15 minutes at room temperature, permeabilized with blocking buffer (PBS with 1% FBS, 0.1% Triton X-100 and 0.1% Tween-20), and stained with RAD51 (Abcam, #ab133534, 1:500 dilution) and Gemini antibodies (Abcam, #ab225396, 1:1000 dilution) for 1 hour at room temperature. The cells were incubated with secondary antibody (Rabbit IgG Alexa 488, Cell Signaling, 4412S 1:3000; Mouse IgG Alexa 555, Cell Signaling Tech, 4409S 1:3000) for 1 hour and washed with PBS. Cells were incubated with Geminin antibody conjugated with Alexa 647 for 1hr. The cells were washed twice with PBS and mounted with DAPI containing Fluor G mounting media and imaged under the Nikon Eclipse 80i microscope. Images were taken with 63x magnification. RAD51 foci per nuclei was counted from >100 Geminin positive cells in 3 fields.

### Patient-derived xenograft (PDX) models

The WO-38, WO-24, WO-93, WO-30, WO-120, WO-28, and WO-36 models were developed by orthotopic transplantation of patient tumor to the ovaries of mice using methods previously described^47^. Patient tumor was obtained from surgeries or biopsies conducted at the Hospital of University of Pennsylvania (IRB approved, # 702679). NOD-SCID IL2Rγ^-/-^ (NSG) mice were purchased from Stem Cell & Xenograft Core at the University of Pennsylvania. All mice were housed according to the policies of the Institutional Animal Care and Use Committee (IACUC) of the University of Pennsylvania (protocol #806002). Once the transplanted tumor tissue reaches approximately 1,000 mm^3^, the mice are euthanized and the tumors are harvested and analyzed by genomic, proteomic, and immunohistochemical studies, expanded by transplanting more NSG mice, and banked in freezing medium (10%DMSO in FBS) for preclinical studies.

For PDX preclinical studies, cryopreserved tissue was thawed, washed with Hank's Balanced Salt Solution, and three chunks (2x2x2 mm) were transplanted to the left distal end of fallopian tube/ovary covered by 100 µl of Matrigel Matrix (Corning Inc., Corning, NY) into 5–8 week-old female NSG mice. The tumor volume was measured weekly using transabdominal ultrasound (SonoSite Edge II Ultrasound System, FUJIFILM SonoSite, Inc, Bothell, WA) by a trained sonographer blinded by treatments information. Tumor length (L) and width (W) were measured and used to calculate tumor volume (volume = L × W2 / 2) for evaluation of *in vivo* drug response. Once tumor volume reached 60 to 100 mm^3^, mice (n=80) were randomized to 7 treatment groups: Control (vehicle; 2-hydroxylpropyl-β-cyclodextrin), carboplatin (Hospira, Inc., Lake Forest, IL; 20 mg/kg for WO-38, WO-30 and WO-120, and 30 mg/kg for WO-24 and WO-93, weekly by intraperitoneal injection), WEE1i (AZD1775 60 mg/kg × 5 days per week by oral gavage), ATRi (AZD6738 40 mg/kg × 5 days per week by oral gavage), BRD4i (AZD5153 1.0–1.5 mg/kg × 5 days per week by oral gavage except WO-24 and WO-93 for 7 days per week), BRD4i+WEE1i (AZD5153 1.0–1.5 mg/kg × 5 days per week by oral gavage except WO-24 and WO-93 for 7 days per week; AZD1775 60 mg/kg × 5 days per week by oral gavage except WO-30 for 45 mg/kg), BRD4i+ATRi (AZD5153 1.0–1.5 mg/kg × 5 days per week by oral gavage except WO-24 and WO-93 for 7 days per week; AZD6738 40 mg/kg × 5 days per week except WO-30 for 30 mg/kg). These dosages and schedules were optimized as maximum tolerable dose by prior dose-de-escalating preliminary studies. The body weights and condition scores of mice were monitored weekly. In all the models, percentage change in body weight during treatment was used as a marker for toxicity and dose level adjustments. Significant treatment toxicity was defined as a 15% drop in body weight and the mice require treatment reduction at 25% dose and supportive supplements care (i.e. gel pack supplement and subcutaneous fluid injection if necessary). For mice with 20% drop in body weight, treatment was stopped, and supportive measures were provided. Body weight was rechecked every 3–4 days. Once improved, treatment was restarted at 25% reduced dose. If body weight was not regained after one week, PDX was euthanized in accordance with IACUC protocols. Trial endpoints were defined as tumor volume > 1000 mm3 or poor condition score (defined as score of 1 on a 1–5-point scale)^75^. Tumors were collected and snap frozen for protein and genomic analysis and fixed in formalin for IHC.

### DNA sequencing of primary tumors and PDX tumors

DNA was extracted by using the Puregene core Kit A (#1042601, Qiagen Inc., Germantown, MD). The DNA samples were fragmented using the Covaris LE220 Focused Ultrasonicator (Covaris, Valencia, CA), Fragmented genomic DNA from cell line samples were used as input for NEBNext Ultra II DNA Library Prep (NEB, Ipswitch, MA) and hybridized using Agilent SureSelect for Target Enrichment with the Agilent Exome Panel v7 (Agilent, Santa Clara, CA) according to the manufacturer’s instructions. Paired-end sequencing resulting in 150 bases from each end of the fragments for whole genome libraries was performed using Illumina NovaSeq 6000 instrumentation (Illumina, San Diego, CA). Human and mouse sequence reads were disambiguated using BBTools^76^. Subsequent reads were aligned to the GRCh37 human reference genome using the Burrows-Wheeler Aligner v0.7.17-r1188^77^. Duplicate reads were marked using Samtools v^78^. Somatic variants were called using Mutect2 following recommended filtering methods^79^.

### Immunohistochemistry

The PDX tumors were collected, fixed with 10% formalin for 24 hours, washed with PBS and maintained in 70% ethanol at 4°C. The tissue samples were dehydrated in graded ethanol, xylene, and embedded in paraffin. Immunohistochemistry of paraffin embedded section (5 µm) was performed using DAKO Envision+System (Dako, Santa Clara, CA). The following primary antibodies were used: PAX8 (Proteintech, Rosemont, IL, #10336-1-AP, 1:1000), ARID1A (Sigma-Aldrich, #HPA005456, 1:500), napsinA (Leica Biosystems Newcastle Ltd, Newcastle upon Tyne, UK, #NCL-L-NapsinA, 1:400), racemase (Zeta Corporation, Arcadia, CA, # Z2001L, 1:50), Ki67 (Dako, #M7240, 1:100), p-Rb (Ser807/811) (Cell Signaling, #8516, 1:200), γH2AX (Ser139) (Cell Signaling, #9718, 1:500), and cleaved caspase-3 (Asp175) (Cell Signaling, #9664, 1:200). Each antibody was incubated for 40 minutes at room temperature. Antigen retrieval for all targets was performed using a pressure cooker in citrate buffer (pH = 6). Appropriate positive and negative (incubation with secondary antibody only) controls were stained in parallel for each round of staining. The percentage of positive cells and staining intensity were reviewed by pathologist and quantified by ImageJ.

### RNA sequencing (RNA-seq)

HCT116 WT and HCT *ARID1A* KO cell lines were treated with vehicle (Control), ATRi (AZD6738) 0.1 µM, BRD4i (AZD5153) 0.1 µM, or combination for 12 hours before collecting the cells. RNA was isolated using the RNeasy Plus Mini Kit (QIAGEN, Venlo, Netherlands, #74134) according to the manufacturer's protocol. The extracted RNA samples were checked for overall quality and submitted to Active Motif, Inc. (Carksbad, CA) for sequencing. RNA-seq and the data processing were performed by Active Motif. Briefly, 42-nt sequence reads were generated by Illumina NextSeq 500. The reads were mapped to the genome using the STAR algorithm with default settings^80^. Fragment assignment was performed by counting the number of fragments overlapping predefined genomic features of interest. Only read pairs that have both ends aligned were counted. Read pairs that have their two ends mapping to different chromosomes or mapping to same chromosome but on different strands were discarded. A minimum of 25 bp overlapping bases was required in a fragment for read assignment. Subread package was used for gene annotations^81^. Genes with the same Entrez gene identifiers were merged into one^81^. Differential gene expression analysis was performed using DESeq2, to identify statistically significant genes^82^. Averages of normalized counts for each group were generated by Affymetrix using DEseq2 pipeline, which filters out genes that have low counts by a statistical technique called independent filtering DEseq2. The DESeq2 model internally corrects for library size using the median-of-ratios method statistics^82^. Log2 fold changes of genes were calculated by DESeq2’s shrinkage (shrunkenLog_2_FC). Differential genes were detected by DESeq2 at 0.1 (or 10%) FDR (Benjamini and Hochberg adjusted p-value) and shrunkenLog2FC cutoff at 0.3. The genes list and the shrunkenLog_2_FCs obtained from DESeq2 were used as an input file to perform Gene Set Enrichment Analysis (GSEA) using the GSEAPreranked tool developed by Broad Institute^83,84^. Pathway enrichment analysis was performed using Reactome Pathway Database^85^.

### Statistics

All statistical analysis was performed using GraphPad Prism 7. For *in vitro* study, Student’s t test was used when comparing two groups and one-way ANOVA was performed for comparisons among >2 groups. For *in vivo* PDX studies, the longitudinal analysis of tumor growth was carried out by linear mixed-effect modeling with type II ANOVA and pairwise comparisons across groups on log pre-processed tumor sizes using the open access TumGrowth web tool (https://kroemerlab.shinyapps.io/TumGrowth/)^86^. Log transformed tumor volume was used to better satisfy normal distribution. Survival data was analyzed by Mantel-Cox log rank test (GraphPad Prism). Adjusted p value < 0.05 was considered statistically significant. * P<0.05, ** P<0.01, *** P<0.001.

## Supplementary Material

Supplement 1
