# Supplementary material for: Dual blockade of BRD4 and ATR/WEE1 pathways exploits ARID1A loss in clear cell ovarian cancer
Source: Res Sq. 2023 Sep 27:rs.3.rs-3314138. Preprint. [Version 1] doi: 10.21203/rs.3.rs-3314138/v1 (PMC10571599; doi:10.21203/rs.3.rs-3314138/v1)
Supplement: Supplement 1 [file NIHPPrs3314138v1-supplement-1.pdf]

# Supplementary Files

This is a list of supplementary files associated with this preprint. Click to download.

- SuppFiguresAndTables083102023.pdf
